# *SWECO25*: a cross-thematic raster database for ecological research in Switzerland

**DOI:** 10.1038/s41597-023-02899-1

**Published:** 2024-01-03

**Authors:** Nathan Külling, Antoine Adde, Fabian Fopp, Anna K. Schweiger, Olivier Broennimann, Pierre-Louis Rey, Gregory Giuliani, Teresa Goicolea, Blaise Petitpierre, Niklaus E. Zimmermann, Loïc Pellissier, Florian Altermatt, Anthony Lehmann, Antoine Guisan

**Affiliations:** 1 EnviroSPACE, Institute for Environmental Sciences, University of Geneva, Geneva, Switzerland; 2 Institute of Earth Surface Dynamics, Faculty of Geosciences and Environment, University of Lausanne, Lausanne, Switzerland; 3 Land Change Science Research Unit, Swiss Federal Institute for Forest, Snow and Landscape Research, WSL, Birmensdorf Switzerland; 4 Ecosystems Landscape Evolution, Institute for Terrestrial Ecosystems, Department of Environmental System Sciences, ETH Zurich, Zurich, Switzerland; 5 Department of Geography, Remote Sensing Laboratories, University of Zurich, Zurich, Switzerland; 6 Department of Land Resources & Environmental Sciences, Montana State University, P.O. Box 173120, Bozeman, MT 597171 USA; 7 Department of Ecology and Evolution, University of Lausanne, Lausanne, Switzerland; 8 GRID-Geneva, Institute for Environmental Sciences, University of Geneva, Geneva, Switzerland; 9 InfoFlora, c/o Conservatoire et Jardin botaniques de Genève, Chambésy-Genève, Switzerland; 10 Department of Evolutionary Biology and Environmental Studies, University of Zurich, Zürich, Switzerland; 11 Department of Aquatic Ecology, Eawag: Swiss Federal Institute of Aquatic Science and Technology, Dübendorf, Switzerland

**Keywords:** Ecology, Environmental sciences, Ecology

## Abstract

Standard and easily accessible cross-thematic spatial databases are key resources in ecological research. In Switzerland, as in many other countries, available data are scattered across computer servers of research institutions and are rarely provided in standard formats (e.g., different extents or projections systems, inconsistent naming conventions). Consequently, their joint use can require heavy data management and geomatic operations. Here, we introduce *SWECO25*, a Swiss-wide raster database at 25-meter resolution gathering 5,265 layers. The 10 environmental categories included in *SWECO25* are: geologic, topographic, bioclimatic, hydrologic, edaphic, land use and cover, population, transportation, vegetation, and remote sensing. *SWECO25* layers were standardized to a common grid sharing the same resolution, extent, and geographic coordinate system. *SWECO25* includes the standardized source data and newly calculated layers, such as those obtained by computing focal or distance statistics. *SWECO25* layers were validated by a data integrity check, and we verified that the standardization procedure had a negligible effect on the output values. *SWECO25* is available on Zenodo and is intended to be updated and extended regularly.

## Background & Summary

Spatial ecology has benefited from growth in data availability, geostatistical methods, and computing facilities, and is now central for a wide range of applications including public health^1–3^, agriculture^4–6^, and biological conservation^7–9^. Because the targets of such applications (e.g., water quality, soil nutrients, species’ environmental suitability) are controlled by multiple environmental drivers (e.g., climate, vegetation, land use and cover), their study requires working with cross-thematic data. However, in most countries, thematic data are often scattered across computer servers of different institutions (e.g., universities, federal and state offices, non-governmental organizations), and are generally lacking spatial standards, whether in terms of resolution, extent, or projection system. Consequently, a significant effort for data compilation and standardization is usually required prior to using them, which implies a non-sustainable use of time and resources, but also hinders comparisons and cross-project usage of data.

In Switzerland, many spatial datasets are publicly available from university and governmental computer servers and cover a wide range of themes including topography^10^, hydrography^11^, land use and cover^12^, transportation^13^, and several others. Although there has been recent efforts for developing web portals aimed at gathering the increasing volume of new data, such as the Swiss Data Cube (https://www.swissdatacube.org/) for remote sensing data^14^, or opendata.swiss (https://opendata.swiss/en) for Swiss government data, available layers are rarely provided in standard formats, so their joint use can require tedious data management and heavy geomatic operations. Currently, a ready-to-use, standard, cross-thematic, geospatial database gathering key layers for ecological research in Switzerland is lacking. Yet, thanks to the large amount of available data and the diversity of landscapes to study and protect, Switzerland is an ideal candidate to promote greater standards in scientific data, which is essential for advancing research in ecology and can inspire similar initiatives worldwide.

Here we introduce *SWECO25*, a 25-meter resolution raster database gathering 5,265 layers on 10 main environmental categories. The 25-meter resolution was chosen as a trade-off between spatial accuracy, resolution of input sources, and size of output database. Layers available in *SWECO25* were standardized to a common spatial grid covering all of Switzerland so they all share the same spatial resolution, extent, and geographic coordinate system. *SWECO25* includes both the standardized sources and newly calculated layers, such as those obtained by computing focal or distance statistics. By providing standardized spatial data for a large range of environmental themes, *SWECO25* stands as a foundational contribution for more effective analyses, informed decision-making, collaboration, and sustainable development across various sectors. It should help streamlining stakeholder workflows and support them in making more accurate decisions. The variety of potential applications of national interest for Switzerland includes, but is not limited to, biodiversity conservation, glacier and snowmelt modelling, tourism and recreation management, natural hazard mitigation, energy transition planning, or ecosystem services assessment. The *SWECO25* database and associated metadata are openly available on Zenodo (https://zenodo.org/communities/sweco25/).

## Methods

**Fig. 1:**
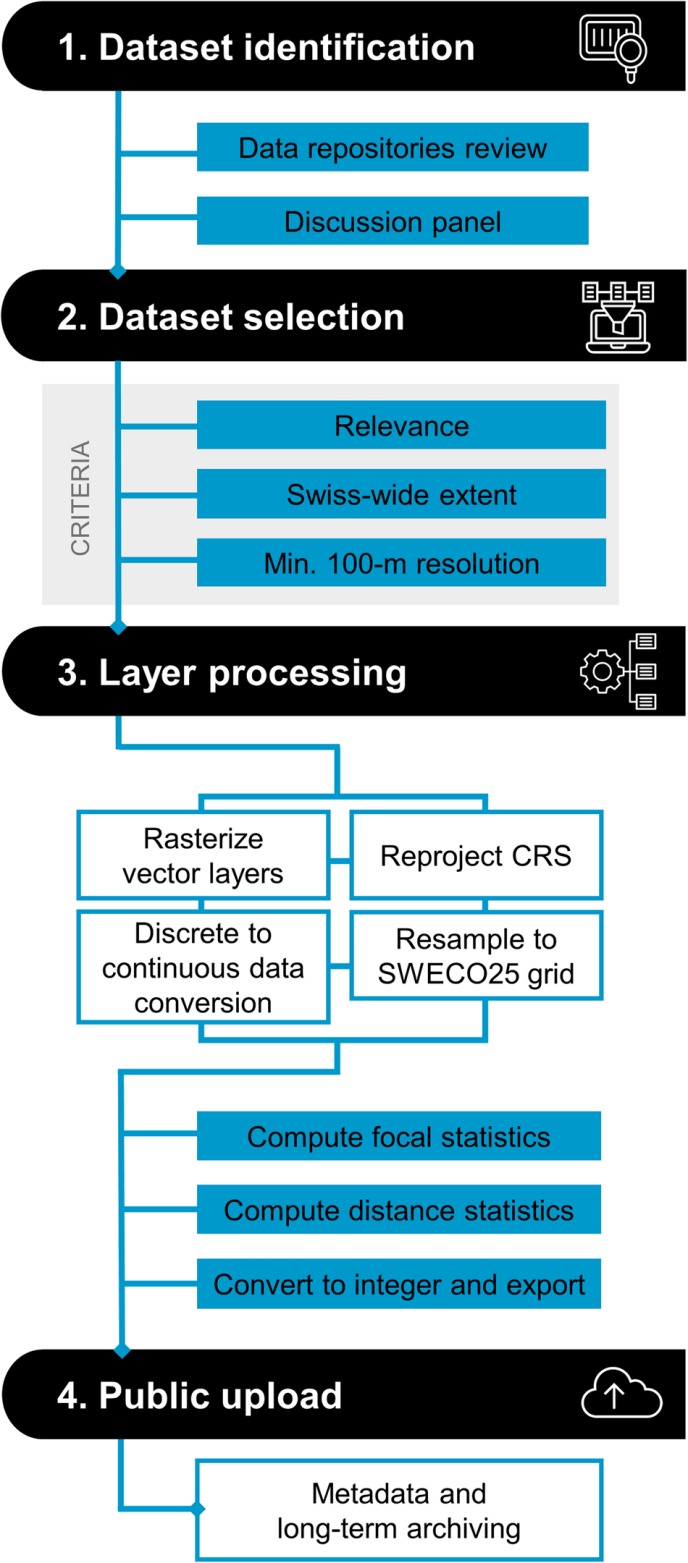
*SWECO25* development workflow. 1) Identification and panel discussion about existing datasets. 2) Selection of ecologically relevant datasets meeting spatial requirements. 3) Standardization of selected datasets to *SWECO25* standards. 4) Public upload on Zenodo (https://zenodo.org/communities/sweco25/).

The development of *SWECO25* followed four main steps (Fig. 1): (1) dataset identification, (2) dataset selection, (3) layer processing, and (4) public upload on Zenodo (https://zenodo.org/communities/sweco25/).

### Dataset identification

We identified candidate datasets for *SWECO25* by screening academic geodata servers (e.g., University of Lausanne UnilGis, Zurich Polytechnic GeoVITe, University of Bern GIUBGIS, and University of Geneva GRID), Swiss governmental geodata (https://opendata.swiss/en), and consulted with a panel of ~20 scientists involved in ecological research in Switzerland. Discussions among panel members allowed sharing unpublished data and identifying gaps in available datasets (e.g., missing themes or coarse spatial resolutions). When these gaps were not solvable by applying basic geomatic operations (e.g., resampling or reprojection), research initiatives involving more advanced computational techniques were started. This was the case for developing the high-resolution climate^15^, and land-use and cover^16^ datasets.

### Dataset selection

Three main criteria were used for deciding on the selection of an existing dataset for *SWECO25*: (i) its relevance for ecological research, (ii) a spatial extent covering all of Switzerland, and (iii) a minimum input resolution of 100 meters to preserve data accuracy after resampling.

### Layer processing

Individual layers from selected datasets were processed following an eight-step standardization procedure: (i) rasterization of vector layers, (ii) reprojection to the CH1903 +/LV95 (https://epsg.io/2056) geographic coordinate system, (iii) resampling to a common spatial grid of 25-meter resolution (bilinear and nearest neighbor methods for continuous and categorical layers, respectively), (iv) transformation of discrete data to continuous values (e.g. converting discrete land use and cover classes within a grid cell to percentage cover for a particular class), (v) computation of distance statistics for linear features (e.g., Euclidean and path distance to roads and rivers), (vi) computation of focal statistics by applying a cell-level function calculating the average value in a circular moving window of 13 radii ranging from 25 meters to 5 kilometers, (vii) conversion of decimals to integer values for storage efficiency, and (viii) saving the final raster layers as GeoTIFF files.

## Data Records

**Fig. 2:**
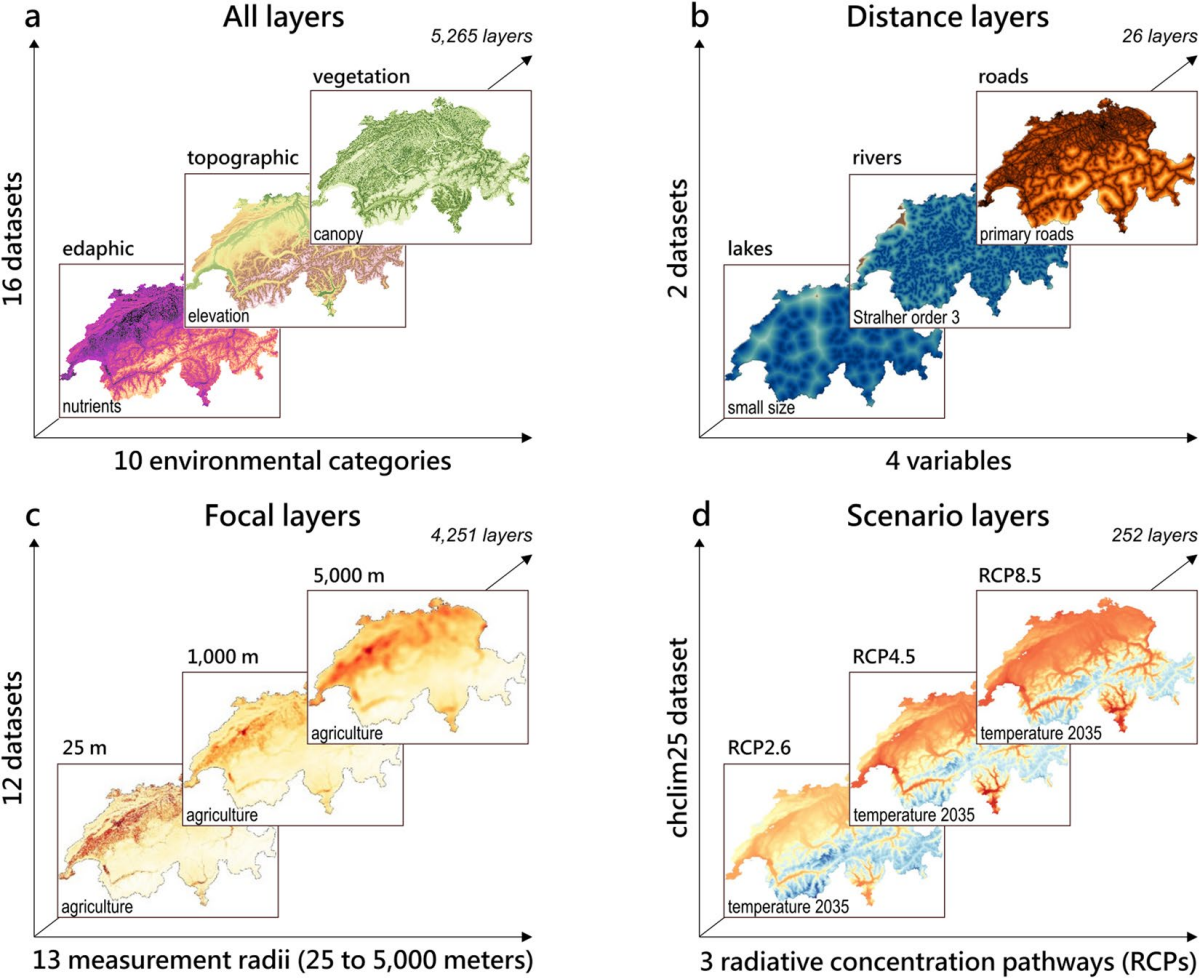
Overview of *SWECO25* layer diversity and example illustrations. (**a**) Example layers extracted from three environmental categories, out of the ten available. (**b**) Example distance statistics layers made available for linear features (i.e., transportation and hydrological networks). (**c**) Example focal statistics layers computed using 13 measurement radii for 12 datasets. (**d**) Example scenarios layers for the chclim25 dataset for three radiative concentration pathways (RCPs).

Following our dataset selection criteria, 16 datasets were collected (Table 1, Table 2, and Table 3). The source datasets consisted predominantly of raster files (10 out of 16) with a mean ± standard deviation (SD) spatial resolution of 32.5 ± 34.8 meters and most of them were already projected in the CH1903 +/LV95 geographic coordinate system (10 out of 16). Most of the datasets were available for a single time step (static), except the chclim25, geostat25, statpop, and sdc datasets that contained layers for several time steps. In addition, the chclim25 dataset included layers for three future greenhouse gas concentration trajectories, or representative concentration pathways (RCPs)^17^, RCP2.6 (“Very Low Carbon”), RCP4.5 (“Low Carbon”), and RCP8.5 (“High Carbon”). After running the standardization procedure, a total of 5,265 layers was produced and compiled together in *SWECO25* (v1.0.0) for a total size of 157 GB. Figure 2 provides an overview of the diversity of layer types available in *SWECO25*.

**Table 1:** Summary characteristics of the bioclimatic, topographic, and geologic datasets included in *SWECO25* (v1.0.0).

Category	Dataset	Short description	Temporal resolution	Input spatial resolution or precision	Number of layers	Reference
Bioclimatic (“bioclim”)	chclim25	Climatic (temperature and precipitation) and bioclimatic WorldClim (https://www.worldclim.org/data/bioclim.html) parameters	Present: annual (1981–2017) and 30-y averages (1981–2010); Future: 30-y averages (2020–49, 2045–70 and 2070–99) for three scenarios (RCP2.6, RCP4.5 and RCP8.5)	25 m (raster)	428	^15^
Topographic (“topo”)	alti3d	Topography without vegetation and development	Static (2016)	2 m (raster)	224	^10^
Geologic (“geol”)	geotechnic	Subsoil classified according to lithological criteria (30 classes)	Static (1967)	0.02 m (vector precision)	420	^30^

**Table 2:** Summary characteristics of the hydrologic, edaphic, and land use and cover datasets included in *SWECO25* (v1.0.0).

Category	Dataset	Short description	Temporal resolution	Input spatial resolution or precision	Number of layers	Reference
Hydrologic (“hydro”)	gwn07	Distance to hydrological network (10 river and 4 lake classes)	Static (2007)	3 to 8 m (vector precision)	14	^11^
Hydrologic (“hydro”)	morph	Ecomorphological state of the rivers (5 classes)	Static (2009)	3 to 8 m (vector precision)	71	^31^
Hydrologic (“hydro”)	swisstopo	Watercourse steepness	Static (2015)	3 to 8 m (vector precision)	28	^32^
Edaphic (“edaph”)	eiv	Ecological indicator values for soil properties	Static (1938–2018)	93 m (raster)	112	^33^
Edaphic (“edaph”)	modiffus	Nitrogen and phosphorus loads	Static (2015)	100 m (raster)	28	^34^
Land use and cover (“lulc”)	geostat25	Land-use and cover classification (65 classes)	6-y periods (1992–1997, 2004–2009 and 2013–2018)	25 m (raster)	2730	^16^
Land use and cover (“lulc”)	wslhabmap	Natural habitats classification (41 classes)	Static (2020)	0.2 to 3 m (vector precision)	574	^35^

**Table 3:** Summary characteristics of the population density, transportation, vegetation, and remote sensing datasets included in *SWECO25* (v1.0.0).

Category	Dataset	Short description	Temporal resolution	Input spatial resolution or precision	Number of layers	Reference
Population density (“pop”)	statpop	Human population density	Annual (2010–2020)	25 m (raster)	297	^36^
Transportation (“trans”)	tlmd3D	Distance to transportation network (5 classes)	Static (2013–2020)	0.2 to 1.5 m (vector precision)	12	^13^
Transportation (“trans”)	sonbase	Exposure to noise levels	Static (2015)	25 m (raster)	14	^37^
Vegetation (“vege”)	nfi	Vegetation height	Static (2019)	10 m (raster)	42	^38^
Vegetation (“vege”)	copernicus	Dominant leaf type	Static (2018)	10 m (raster)	28	^39^
Remote sensing indices (“rs”)	sdc	Remote sensing indices (EVI, GCI, LAI, NDVI, NDWI)	Annual (1996–2021)	10 m (raster)	243	^40^

**Fig. 3:**
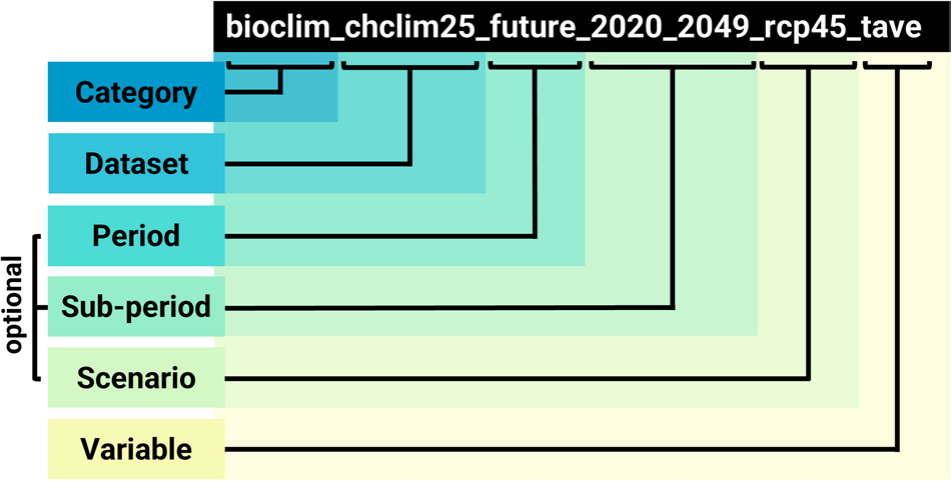
*SWECO25* folder and file naming structure. In this example, the “tave” (temperature average) variable, from the “bioclim” (bioclimatic) category, in the “chclim25” dataset, for the “future” period, “2020_2049” sub period, and the “rcp45” scenario is stored in the folder “bioclim/chclim25/future/2020_2049/rcp45/tave”. The filename for this variable is “bioclim_chclim25_future_2020_2049_rcp45_tave.tif”.

All *SWECO25* layers and files are following a standard naming scheme, which is also used for folder organization (Fig. 3). The tree structure of *SWECO25* folders can be developed to a maximum of six levels: category, dataset, period, sub-period, scenario, and variable (Fig. 3). At its top level *SWECO25* is divided into ten main environmental categories: geologic (“geol”)^18^, topographic (“topo”)^19^, bioclimatic (“bioclim”)^20^, hydrologic (“hydro”)^21^, edaphic (“edaph”)^22^, land use and cover (“lulc”)^23^, population (“pop”)^24^, transportation (“trans”)^25^, vegetation (“vege”)^26^, and remote sensing indices (“rs”)^27^. The environmental category with the most layers was land use and cover (3,304), followed by bioclimatic (428) and geologic (420). For each environmental category, the detailed list of layers can be found in the SWECO25_datalayers_details_categoryname.csv file available in its respective Zenodo repository.

## Technical Validation

*SWECO25* was technically validated through an automated procedure during which all layers were checked for their standard format (reference system, spatial resolution, and extent), folder structure, naming scheme, and data integrity (count of NA cells, range of values, and integer format). All layers successfully passed the validation procedure, except for several from the remote sensing category that contained a higher number of NA cells, as well as very high or low values. The propagation of errors inherent to the source data to *SWECO25* is difficult to avoid and to measure. For instance, reason for NA cells is most likely artefacts from satellite imagery, whereas very high or low values arise because remote sensing vegetation indices were computed for all of Switzerland, including areas not covered by vegetation. We did not mask very high and low values, as they might still be informative for some users (e.g. provide information on the presence or absence of vegetation). The supplementary file SWECO25_datalayers_details_rs.csv available on the *SWECO25* remote sensing Zenodo repository (https://zenodo.org/record/7994481) allows identifying these layers. In addition, we assessed the potential effects of spatial resampling on *SWECO25* layers by comparing the values of 15'000 random points extracted from the source and resampled layers. This analysis was conducted for source layers from all datasets, except the gwn07 and tlmd3D datasets that consisted of linear features (i.e., river and road networks, respectively) from which distance layers were computed directly on the *SWECO25* grid. The chclim25 dataset was also excluded from this analysis as the source dataset used the *SWECO25* grid. For the “sdc” dataset, due to the number of source layers in it, only three time-steps were randomly tested for each spectral index. Results from the resampling analysis indicated very low differences between source and resampled values, with a median coefficient of variation^28^ ± standard deviation of 0.0066 ± 0.0348 for continuous data (32 source layers evaluated), and a median Dice coefficient^29^ ± standard deviation of 0.9906 ± 0.0793 for discrete data (7 source layers evaluated). A detailed version of the technical validation procedure with additional results can be found on the *SWECO25* GitHub repository (https://github.com/NKulling/SWECO25/tree/main/database_validation).

## Data Availability

The R-code and ArcGIS toolboxes allowing to reproduce the standardization procedure, the computation of focal and distance statistics, and the technical validation are openly available on the *SWECO25* GitHub repository https://github.com/NKulling/SWECO25.
